# Development of tools to facilitate the diagnosis of hereditary fructose intolerance

**DOI:** 10.1002/jmd2.12379

**Published:** 2023-07-27

**Authors:** Bianca Panis, Lise E. F. Janssen, Dirk J. Lefeber, Nynke Simons, M. Estela Rubio‐Gozalbo, Martijn C. G. J. Brouwers

**Affiliations:** ^1^ Division of Genetic Metabolic Diseases, Department of Pediatrics Maastricht University Medical Center Maastricht The Netherlands; ^2^ Member of European Reference Network for Hereditary Metabolic Diseases (MetabERN); ^3^ Member of United for Metabolic Diseases (UMD); ^4^ Division of Endocrinology and Metabolic Diseases, Department of Internal Medicine Maastricht University Medical Center Maastricht The Netherlands; ^5^ Translational Metabolic Laboratory, Department of Laboratory Medicine Radboud University Medical Center Nijmegen The Netherlands; ^6^ Department of Neurology Radboud University Medical Center Nijmegen The Netherlands; ^7^ Laboratory for Metabolism and Vascular Medicine, Division of General Internal Medicine, department of Internal Medicine Maastricht University Medical Center Maastricht The Netherlands; ^8^ CARIM, School for Cardiovascular Diseases Maastricht The Netherlands; ^9^ Department of Clinical Genetics Maastricht University Medical Center, Maastricht University Maastricht The Netherlands; ^10^ GROW‐School for Oncology and Developmental Biology, Faculty of Health, Medicine and Life Sciences Maastricht University Maastricht The Netherlands

**Keywords:** food diary, fructose, glycosylation of transferrin, hereditary fructose intolerance (HFI)

## Abstract

Although hereditary fructose intolerance (HFI) is an inborn error of fructose metabolism that classically presents at infancy, the diagnosis is often missed or delayed. In this study, we aimed to develop tools to facilitate the diagnosis of HFI. The intake of fructose‐containing food products, that is, fruit, fruit juice and sugar‐sweetened beverages, was assessed by a 3‐day food diary in adult HFI patients (*n* = 15) and age, sex, and BMI‐matched controls (*n* = 15). Furthermore, glycosylation of transferrin was examined using high‐resolution mass spectrometry and abnormally glycosylated transferrin was expressed as ratio of normal glycosylated transferrin. We found that the sensitivity and specificity of the 3‐day food diary for the intake of at least one fructose‐containing food product were both 100%. Both mono‐glyco:diglyco transferrin and a‐glyco+mono‐glyco:di‐glyco transferrin were greater in HFI patients and had a high‐discriminatory power (area under the receiver operating characteristic curve: 0.97 and 0.94, respectively). In this well‐characterized cohort of adult HFI patients, the 3‐day food questionnaire and the glycosylation pattern of transferrin are valuable tools to facilitate the recognition and diagnosis of HFI in adult patients.

**SYNOPSIS**

A 3‐day food questionnaire and the glycosylation pattern of transferrin are valuable tools to facilitate the recognition and diagnosis of HFI in adult patients.

## 1 INTRODUCTION

Hereditary fructose intolerance (HFI; OMIM 229600) is an inborn error of fructose metabolism caused by biallelic pathogenic variants in the gene encoding aldolase B (*ALDOB; EC 4.1.2.13*).^1^, ^2^ Although HFI typically manifests in children during weaning when fruits and vegetables are introduced, there is anecdotal evidence that the diagnosis is not always recognized or that parents do not seek medical attention.^3^ Based on the discrepancy between the estimated prevalence, that is, 1:10 000, which would render HFI one of the most prevalent inborn errors of metabolism,^4^ and the real life prevalence at the metabolic centers, where HFI is not present in the list of most frequent diagnoses,^5^ it appears that the number of undiagnosed patients with HFI can be substantial.

In order to facilitate the recognition and diagnosis of HFI, it is desirable to have diagnostic tools that can be easily used as a screening instrument, such as questionnaires and biomarkers. The typical history, that is, strong aversion against fructose‐containing food products, could be implemented in a diagnostic questionnaire, but has never been tested.

**FIGURE 1:**
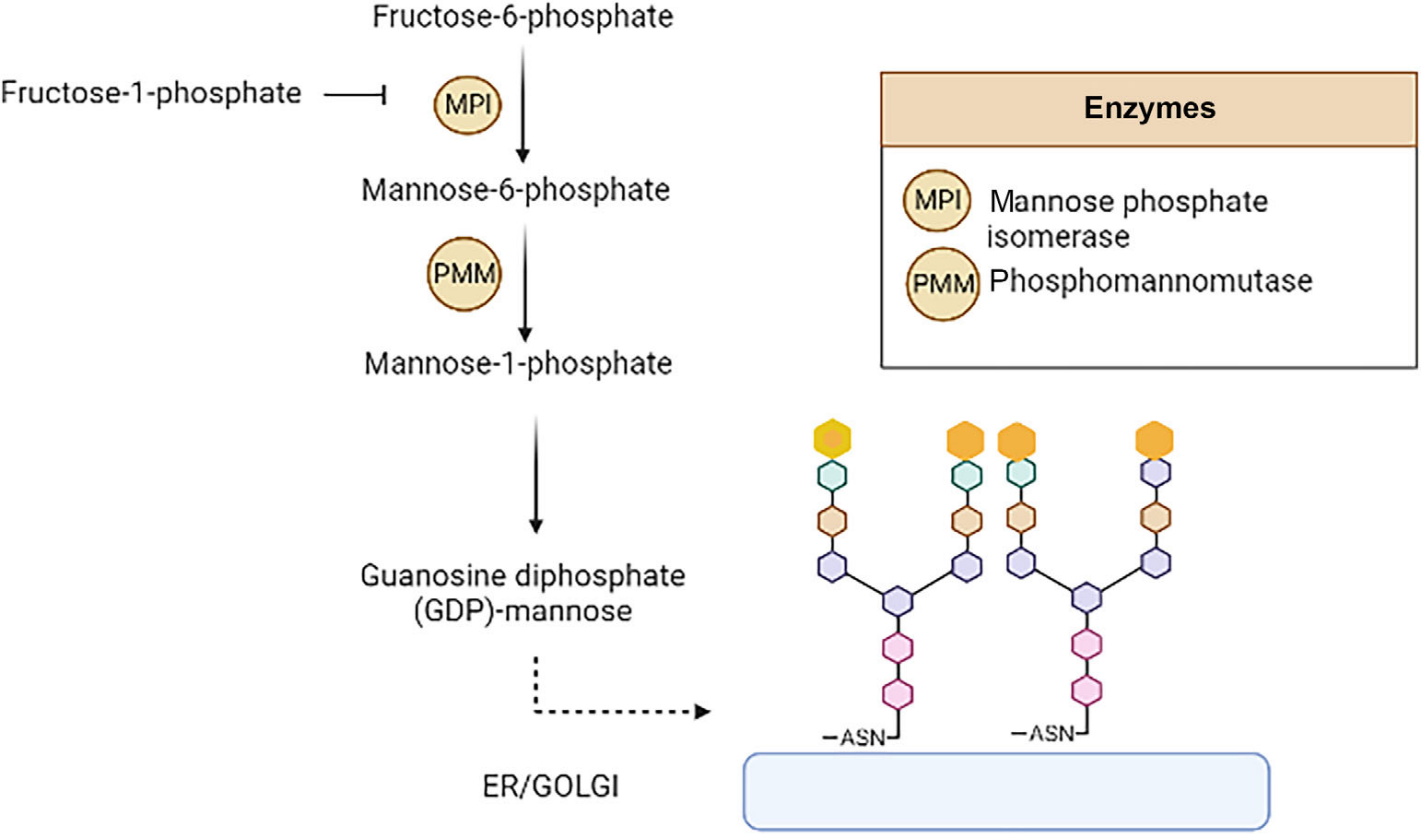
Normal transferrin consists of two sialylated bi‐antennary complex glycans attached to the two glycosylation sites of the protein (diglycosylated transferrin). Suppression of transferrin glycosylation via the competitive inhibition of mannose phosphate isomerase (MPI) by fructose‐1‐phophate results in the loss of one or both antennary structures (a‐glycosylated transferrin, monoglycosylated transferrin).

Furthermore, an abnormal transferrin glycosylation pattern, which has been described in untreated patients with HFI, could serve as a useful diagnostic biomarker.^6^, ^7^ Normal transferrin consists of two sialylated bi‐antennary complex glycans attached to the two glycosylation sites of the protein (diglycosylated transferrin). Suppression of transferrin glycosylation via the competitive inhibition of mannose phosphate isomerase (MPI) by fructose‐1‐phophate results in the loss of one or both antennary structures (Figure 1).^8^ By using high‐resolution mass spectroscopy, we recently showed that dietary treated, adult HFI patients are still characterized by more hypoglycosyated transferrin than age‐ and sex matched controls, albeit within the normal range.^9^ This may be explained by trace amounts of (hidden) fructose in (non‐) foods.^10^

In this study we aimed to develop tools to facilitate the diagnosis of HFI. For this, we assessed whether a short questionnaire and the glycosylation pattern of transferrin could serve as valuable diagnostic markers of HFI, even in patients with self‐imposed fructose restriction.

## 2 METHODS

### 2.1 Participants

Fifteen adult HFI patients were recruited from adult metabolic centers in the Netherlands and Belgium and compared with healthy individuals matched for age, sex, and BMI, as previously described.^9^ Diagnosis of HFI was confirmed by a fructose tolerance test in 1, measurement of aldolase activity in liver biopsy tissue in 2, and by genetic analysis in 12 patients. Healthy individuals were recruited via local advertisements in the vicinity of Maastricht.

All participants gave written informed consent before inclusion in the study. The study was performed according to the Declaration of Helsinki and approved by the medical ethical committee of Maastricht University/Academic Hospital Maastricht (METC 162003).^11^

### 2.2 Assessment of dietary intake of fructose

All participants were asked to complete a 3‐day food journal, which was combined with a personal interview with the researcher (NS). The calculation of daily fructose intake was based on an extensive sugar composition table.^9^, ^12^ Since fruits, fruit juices and sugar‐sweetened beverages are a major source of daily fructose intake,^12^ we tested the discriminative power of these fructose‐containing food products to diagnose HFI.

### 2.3 Glycosylated transferrin

Measurement of transferrin glycosylation patterns was carried out by using high‐resolution mass spectrometry.^13^ Two μL of eluted sample was analyzed on a microfluidic 6540 high‐performance liquid chromatography/chip quadrupole time‐of‐flight instrument (Agilent Technologies, Santa Clara, CA). Data analysis was performed by using Agilent Mass Hunter Qualitative Analysis software B.04.00. Agilent BioConfirm software was used to deconvolute the charge distribution raw data to reconstructed mass data. If no monoglycosylated transferrin was present in the samples, abundances were estimated at the average reconstructed masses of 77 351 Da.

In this study, the transferrin glycosylation pattern was expressed as the a‐glycosylated transferrin, monoglycosylated transferrin, a‐glycosylated+monoglycosylated transferrin, and trisialo‐transferrin isoforms, all as ratio of diglycosylated transferrin (a‐glyco:di‐glyco transferrin, mono‐glyco:di‐glyco transferrin, a‐glyco+mono‐glyco:di‐glyco transferrin, and trisialo:di‐glyco‐transferrin, respectively).

### 2.4 Statistical analyses

Continuous variables are presented as medians with interquartile range. A Mann–Whitney *U* test was used to compare HFI patients with controls.

Sensitivity and specificity were calculated for the daily and 3‐day intake of fruits, fruit juices or sugar‐sweetened beverages (yes or no) as a diagnostic criterion of HFI.

The discriminatory power of hypoglycosylated transferrin was assessed by calculating the area under the receiver operating characteristic (AUROC) curve. The optimal score cut‐off was derived when the sum of sensitivity and specificity was maximum.

Results were considered statistically significant at *p* < 0.05. All analyses were carried out with the SPSS software, version 28 for Windows (IBM, Chicago, IL).

## 3 RESULTS

### 3.1 General characteristics

General characteristics of the 15 HFI patients and healthy participants are displayed in Table 1. Age, sex, and BMI were, by design, not different between both groups. As expected, daily fructose intake was very low in HFI patients (*p* < 0.05).

**TABLE 1:** Characteristics of the study population.

Characteristics	Healthy controls	HFI patients
Men (*n*)/women (*n*)	11/4	11/4
Age (years)	28 (25–52)	31 (24–44)
BMI (kg/m^2^)	21.8 (21.0–23.3)	20.4 (19.3–24.8)
Fructose intake, g/day^a^	36.0 (23.7–57.50)	1.0 (0.8–1.5)^#^
Alcohol intake, U/day^a^	0.6 (0.1–1.7)	0.1 (0.0–0.3)^#^

*Note*: Data are expressed as median (interquartile range).

^a^ Available for 14 HFI patients and 13 healthy controls.

^#^ *p* < 0.05, Mann–Whitney *U* test.

### 3.2 Discriminative power of intake of fruit, fruit juices, and sugar‐sweetened beverages to diagnose hereditary fructose intolerance

Fourteen of the 15 HFI patients completed the 3‐day food diary. Of the healthy controls, 15 completed the first day, 14 the first and second day, and 13 all 3 days.

Analyses of the 3‐day food diaries showed that none of the 14 HFI patients consumed fruits or fruit juices, whereas 11 of the 13 healthy controls consumed at least one piece of fruit or one glass of fruit juice during the 3‐day food diary. Furthermore, none of the HFI patients consumed sugar‐sweetened beverages, versus eight healthy controls, who consumed at least one glass of sugar‐sweetened beverage during the 3‐day food diary.

The sensitivity and specificity of the intake of any fruits, fruit juice or sugar‐sweetened beverages for the diagnosis of HFI based on the 3‐day food diary was both 100% (Table 2).

**TABLE 2:** Sensitivity and specificity for the diagnosis of HFI, based on the intake of any fruits, fruit juices, and sugar‐sweetened beverages according to the 3‐day food diary and according to the separate days.

	HFI patients	Healthy controls	Total
Three‐day food diary			
Positive (*n*)^a^	14	0	14
Negative (*n*)^b^	0	13	13
Total (*n*)	14	13	27
	Sensitivity = 100%	Specificity = 100%	
Day 1 of the 3‐day food diary			
	HFI patients	Healthy controls	Total
Positive (*n*)^a^	14	0	14
Negative (*n*)^b^	0	15	15
Total (*n*)	14	15	29
	Sensitivity = 100%	Specificity = 100%	
Day 2 of the 3‐day food diary			
	HFI patients	Healthy controls	Total
Positive (*n*)^a^	14	3	17
Negative (*n*)^b^	0	11	11
Total (*n*)	14	14	28
	Sensitivity = 100%	Specificity = 78.6%	
Day 3 of the 3‐day food diary			
	HFI patients	Healthy controls	Total
Positive (*n*)^a^	14	3	17
Negative (*n*)^b^	0	10	10
Total (*n*)	14	13	27
	Sensitivity = 100%	Specificity = 76.9%	

^a^ Test is regarded positive when no fruits, fruit juices, and sugar‐sweetened beverages have been consumed.

^b^ Test is regarded negative when fruits, fruit juices, or sugar‐sweetened beverages have been consumed.

The sensitivity of the intake of any fruits, fruit juice or sugar‐sweetened beverages for the diagnosis of HFI based on a single day food diary was 100% for all three single days and the specificity 100%, 78.6%, and 76.9% for the first, second, and third day, respectively (Table 2).

### 3.3 Discriminatory power of transferrin glycosylation patterns

Mono‐glyco:di‐glyco transferrin and a‐glyco+mono‐glyco:di‐glyco transferrin were more abundant in dietary treated HFI patients in comparison to healthy controls (*p* < 0.001), whereas the opposite was found for the ratio trisialo:di‐glyco‐transferrin (*p* = 0.002) (Table 3).

**TABLE 3:** Glycosylation pattern of transferrin in HFI patients and controls. Data are expressed as median (interquartile range).

	Healthy controls	HFI patients	*p*
A‐glyco:di‐glyco transferrin	0.0 (0.0–0.004)	0.0 (0.0–0.02)	0.25
Mono‐glyco:di‐glyco transferrin	0.02 (0.02–0.03)	0.08 (0.06–0.13)	< 0.001
A‐glyco+mono‐glyco:di‐glyco transferrin	0.02 (0.02–0.03)	0.08 (0.07–0.14)	< 0.001
Trisialo:di‐glyco‐transferrin	0.06 (0.05–0.07)	0.05 (0.03–0.06)	0.002

*Note*: Analyzed with Mann–Whitney *U* test.

**FIGURE 2:**
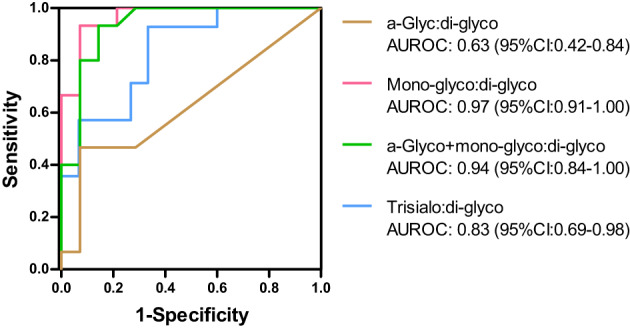
The discriminative power of the transferrin glycosylation patterns.

The discriminative power of the transferrin glycosylation patterns is depicted in Figure 2. The ratio of mono‐glyco:di‐glyco transferrin had an AUROC curve of 0.97 (95% CI: 0.91–1.00). The sensitivity and specificity were 93.3% and 92.9%, respectively, at a cut‐off of 0.052. The ratio of a‐glyco+mono‐glyco:di‐glyco transferrin had an AUROC curve of 0.94 (95% CI: 0.84–1.00) with a sensitivity of 93.3% and specificity of 85.7%, at a cut‐ off point of 0.045. The ratio of a‐glyco:di‐glyco transferrin had an AUROC curve of 0.63 (95% CI: 0.42–0.84) with a sensitivity of 46.7% and specificity of 92.9%, at a cut‐ off point of 0.007. Finally, the ratio of trisialo:di‐glyco‐transferrin had an AUROC curve of 0.83 (95% CI: 0.69–0.98) with a sensitivity of 92.9% and specificity of 66.7%, at a cut‐off point of 0.050 .

## 4 DISCUSSION

The present study was conducted to evaluate whether easily obtainable questionnaires and serum biomarkers can serve as diagnostic tools to facilitate the diagnosis of HFI. We found that a 3‐day questionnaire based on the intake of fruit, fruit juice, and sugar‐sweetened beverages has optimal test characteristics to diagnose HFI. In addition, the discriminative power of transferrin glycosylation patterns, in particular the mono‐glyco:di‐glyco ratio, was good as well, even in dietary treated patients. The usability of these tools to screen in the general population for HFI, or in selected groups of patients, should now be investigated.

We hypothesized that the intake of fruits, fruit juices and sugar‐sweetened beverages are potentially good instruments to diagnose HFI, since they are high in fructose content and are commonly consumed. Indeed, we observed that a 3‐day food questionnaire, based on the intake of any fruits, fruit juice or sugar‐sweetened beverages, perfectly discriminated HFI from non‐HFI. It should be noted that the sensitivity could have been inflated as a consequence of dietary counseling, since these patients have been instructed to avoid these food products. However, in our experience most patients have learnt to avoid any fructose‐containing food, particularly fruits and sugar‐sweetened beverages, by trial and error, independent of dietary counseling. The high specificity as found in this study, depends on the amount of fructose consumed in the general population. The higher the consumption, the higher the specificity. In the Netherlands, the adult median habitual fructose intake is 46 g/day,^12^ which is lower in comparison to previous estimates in the United States (54 g/day).^14^ The highest intake is observed among adolescents (63 and 52 g/day for Dutch boys and girls, respectively, and 73 g/day for both boys and girls in the United States).^12^, ^14^

Previous studies have shown that abnormal transferrin glycosylation patterns are found in untreated patients with HFI.^6^, ^7^, ^15^, ^16^, ^17^ We recently showed that the ratio mono‐glyco:di‐glyco transferrin was higher, albeit within the normal range, in dietary treated HFI patients in comparison to the healthy controls.^9^ These results are not explained by chronic alcohol consumption, which is known to affect glycosylation,^18^ as alcohol intake was low in patients with HFI. In this study, we found that the ratio trisialo:di‐glyco was also statistically significantly different between both groups. The highest discriminative power to distinguish HFI from non‐HFI was found for mono‐glyco:di‐glyco transferrin with an AUROC curve of 0.97 (sensitivity of 93.3% and specificity of 92.9%, at a cut‐off level of 0.052). Our results are in line with those from Cano and colleagues who measured glycosylation pattern using capillary zone electrophoresis. They concluded that the tetrasialo‐transferrin:disialo‐transferrin ratio, which corresponds to the inverse of mono‐glyco:di‐glyco transferrin in our study, differentiated dietary treated HFI patients from healthy controls with an AUROC curve of 0.97.^19^

In this well‐characterized cohort of adult HFI patients, both the food questionnaire and the transferrin glycosylation pattern had high discriminative power to distinguish HFI from non‐HFI patients, which makes them suitable instruments to screen for HFI in selected populations, e.g. in children with presumed food allergies, avoidant restrictive food intake disorder (ARFID), and/or unexplained abdominal complaints and liver disease. It is expected that there are yet undiagnosed HFI cases among these populations. Indeed, a case of HFI was recently identified by whole exome sequencing among lean, adult patients with non‐alcoholic fatty liver disease.^20^ However, before these tools can be implemented in clinical practice, they need to be replicated and validated. As hitherto mentioned, it is not clear whether the diagnostic performance of the questionnaire is similar in children/adolescents and/or patients who have not yet received dietary counseling. Although our findings for the transferrin glycosylation pattern can be viewed as a replication of the recent study by Cano and colleagues, who included both adult *and* non‐adult patients with HFI,^19^ transferrin glycosylation was assessed by different methods which make a definite conclusion regarding the optimal diagnostic cut‐off value difficult.

## 5 CONCLUSION

In this well‐characterized cohort of adult HFI patients, the 3‐day food questionnaire and the transferrin glycosylation pattern showed a high discriminative power to discern HFI from non‐HFI. Further studies are needed to replicate and validate these findings in order to use these tools as screening instruments to facilitate the diagnosis of HFI in selected patient groups.

## AUTHOR CONTRIBUTIONS

Bianca Panis: Contribution: contributed to pertinent aspects of the planning, conduct, and reporting of the work, conducted and analyzed the analyses, researched the data and wrote the manuscript. Guarantor: Martijn Brouwers.

Lise E.F. Janssen: Contribution: performed the measurements and interpreted the data and reviewed the manuscript. Guarantor: Martijn Brouwers.

Dirk J. Lefeber: Contribution: conducted and analyzed the glycoprofiling of transferrin, reviewed the manuscript, and provided revisions to the manuscript. Guarantor: Martijn Brouwers.

Nynke Simons: Contribution: performed and analyzed the measurements and reviewed the manuscript. Guarantor: Martijn Brouwers.

M. Estela Rubio‐Gozalbo: Contribution: Involved in conception, and reviewed the manuscript, and provided revisions to the manuscript. Guarantor: Martijn Brouwers.

Martijn C.G.J. Brouwers: Contribution: He is involved in the design of the study. He is the guarantor of this work and as such, had full access to all the data in the study and takes responsibility for the integrity of the data and the accuracy of the data analysis and controlled the decision to publish. He contributed to pertinent aspects of the planning, conduct, and reporting of the work.

## FUNDING INFORMATION

This study was supported by research grants from Stofwisselkracht and the Netherlands Heart Foundation (grant 2015 T042; M.B.).

## CONFLICT OF INTEREST STATEMENT

Bianca Panis, Lise E.F. Janssen, Dirk J. Lefeber, Nynke Simons and M. Estela Rubio‐Gozalbo declare that they have no conflict of interest. Martijn C.G.J. Brouwers received consultancy fees from Arrowhead and Editas Medicine.

## INFORMED CONSENT

All procedures followed were in accordance with the ethical standards of the responsible committee on human experimentation (institutional and national) and with the Helsinki Declaration of 1975, as revised in 2000. Informed consent was obtained from all patients for being included in the study.

Proof that informed consent was obtained is available upon request.

## ETHICS STATEMENT

This study was approved by the medical ethical committee of Maastricht University/Academic Hospital Maastricht (METC 162003).

## Data Availability

The data that support the findings of this study are available from the corresponding author upon reasonable request.
